# From forest to frontline: A comprehensive review of Mpox's global leap and viral evolution (2022–2024)

**DOI:** 10.1016/j.onehlt.2025.101232

**Published:** 2025-10-03

**Authors:** Baylor Akhavan, Abu-Bakr Ahmed, Wilson To, Samir Alkhouri, Kavita Batra

**Affiliations:** a Kirk Kerkorian School of Medicine at UNLV, 625 Shadow Lane, Las Vegas, NV 89106, United States; b Department of Medical Education and Office of Research, Kirk Kerkorian School of Medicine at UNLV, 1701 W. Charleston Blvd., Las Vegas, NV 89106, United States

**Keywords:** Mpox (MPXV), Clade I, Clade II, APOBEC3 mutations, Pediatric mortality, Public health surveillance

## Abstract

**Background:**

Since 2022, the global epidemiology of Mpox (MPXV) has transformed, with Clade IIb driving widespread transmission in non-endemic regions and Clade I resurging across Central Africa. Genomic adaptations, such as APOBEC3 mutations, and increasing human-to-human transmission have raised public health alarms. This review synthesizes the latest evidence on Clade I and Clade II MPXV outbreaks from 2022 to 2024, with an emphasis on geographic expansion, demographic disparities, and systemic response gaps.

**Methods:**

A comprehensive literature search was conducted between September and December 2024 using PubMed, CDC, WHO, and ECDC databases. Studies were included if they reported primary data on Clade I or Clade II MPXV epidemiology. Gray literature, including public health reports and situation summaries, was also analyzed. Data extraction focused on demographic trends, transmission modes, genomic mutations, and public health responses.

**Results:**

Fifty-one sources were reviewed, including 14 peer-reviewed articles and 37 Gy literature reports. Clade IIb accounted for 117,000+ cases across 123 countries, primarily affecting men who have sex with men (MSM). Clade I resurged in the Democratic Republic of the Congo (DRC) with over 19,000 confirmed cases and 975 deaths, disproportionately affecting children under five. APOBEC3-type mutations were linked to increased Clade I transmissibility. International spread of Clade I was reported in the U.S., Europe, and Asia. Diagnostic limitations, especially in the DRC, and gaps in pediatric vaccine access remain major challenges.

**Conclusion:**

MPXV's global trajectory now includes sustained transmission of both Clades I and II. Expanded genomic surveillance, pediatric-focused outbreak response, and equitable diagnostic and vaccine access are urgently needed. A One Health approach is essential to address evolving viral behavior and health disparities in endemic and non-endemic regions.

## Highlights

• Clade IIb remained dominant in global spread, especially among MSM populations.
• Clade I Mpox resurged in 2024 with sustained human transmission across Central Africa.
• Genomic data revealed APOBEC3 mutations linked to Clade I transmission shifts.
• Children under five faced the highest mortality rates in Clade I outbreaks in the DRC.
• Diagnostic access remained below 20 % in the DRC, and children lacked adequate vaccine coverage and treatment access.

## Graphical abstract

**Figure:**
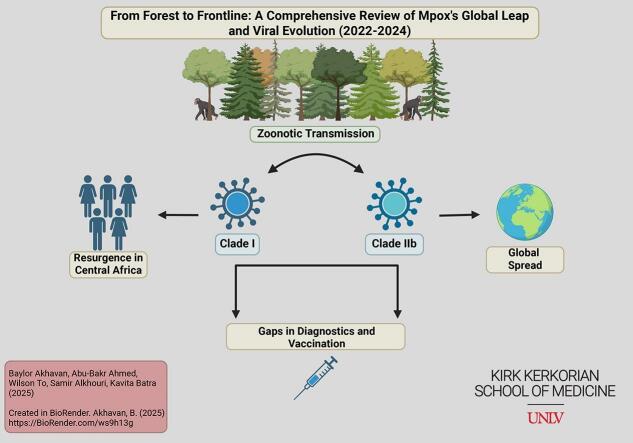

## 1 Introduction

In May 2022, Mpox virus (MPXV) outbreaks emerged in clusters across Europe and North America. This event sparked global attention and marked multiple international outbreaks for a virus previously endemic to Central and West Africa. MPXV is a zoonotic member of the *Poxviridae* family and *Orthopoxvirus* genus first identified in monkey studies in a Danish laboratory in 1958 [1]. The first documented diagnosis of human MPXV infection occurred in the Democratic Republic of the Congo (DRC) [2]. Since then, this disease broke out through the DRC, spread throughout multiple countries in Central and West Africa over the latter half of the 20th century, and first emerged outside of Africa in 2003 in a case linked to infected African rodents that infected pet prairie dogs [3,4].

MPXV is genetically divided between two distinct clades: Clade I and Clade II, both of which differ in their virulence and epidemiological patterns. Both clades typically demonstrate a prodromal phase characterized by fever, lymphadenopathy, and myalgias followed by a progressive maculopapular rash within one and four days before or after initial systemic illness, though some cases have demonstrated this rash without associated systemic illness [5]. Clade I infections are distinguished clinically by their severity and systemic involvement, with case fatality rates around 10 %. Clade II has demonstrated milder symptoms and lower-case fatality rates at 3.6 % [4]. Both clades of MPXV have historically been transmitted through direct contact with infected animals or contact with contaminated blood, fluid, or respiratory droplets from a host. However, the global outbreak in 2022 marked a pivotal change in MPXV epidemiology when over 100 previously non-endemic countries began reporting widespread human-to-human transmission driven primarily by MPXV's Clade IIb lineage [6,7]. Ongoing efforts in the medical and scientific community have been made to characterize changes in MPXV transmission routes, identify high-risk populations, and assess the effectiveness of public health responses to contain the virus such as vaccination programs [8]. Epidemiological patterns characterizing the evolving characteristics of MPXV up to 2022 are well-documented, however, there appear to be gaps in extant peer-reviewed literature describing its most recent progression between the 2022 outbreak and 2024. Characterizing the evolving epidemiological patterns of MPXV can offer insights into disease prevention strategies and inform global public health response.

While prior reviews have examined various dimensions of the 2022–2024 Mpox outbreaks, including clinical presentation, transmission, and diagnostic strategies, they often prioritize biomedical outcomes or regulatory frameworks. For instance, Yadav et al. (2025) provides a comprehensive synthesis of complications, diagnostic developments, and treatment approaches across multiple regions, while Tiwari et al. (2025) offers an in-depth examination of global regulatory responses, public health infrastructure, and policy-level challenges during the Mpox response [9,10]. Other recent genomic reviews have focused on viral mutation dynamics, including the role of APOBEC3-mediated evolution in Clade I and IIb strains, which may impact transmissibility and vaccine response. While these studies make important contributions, our review offers a distinct perspective by integrating surveillance data and outbreak summaries with a specific emphasis on demographic disparities, particularly the disproportionate pediatric burden in Central Africa and gaps in diagnostic access. In doing so, we aim to complement existing literature by offering an equity- and surveillance-centered synthesis that highlights both the biological evolution of MPXV and the systemic vulnerabilities shaping its global trajectory. This approach aligns with a One Health framework advanced in earlier outbreak response tools, such as the Identify–Isolate–Inform (3I) model developed by Koenig et al. (2022) and later adapted by Marty et al. (2024) in response to global Clade IIb outbreaks [11,12]. Furthermore, this review will characterize the differences between the major extant clades of MPXV and highlight data gaps for future exploration.

## 2 Methods

A comprehensive literature search was conducted from September 2024 to December 2024 to identify peer-reviewed studies and relevant gray literature. This review examined extant peer-reviewed literature and gray literature including public health reports, policy/executive documents, and outbreak summaries from global health organizations. Additionally, the definition of gray literature was outlined as research and information produced by organizations outside of traditional commercial or academic publishing channels. This includes materials not controlled by commercial publishers or typically disseminated for specific audiences, with examples including government reports, policy documents, white papers, conference proceedings, theses, and technical reports. These sources can provide insights that may not yet be adequately characterized in peer-reviewed literature in light of the evolving nature of MPXV outbreaks. Searches were performed using databases such as PubMed, the World Health Organization (WHO), the Centers for Disease Control and Prevention (CDC), and the European Centre for Disease Prevention and Control (ECDC). The search focused on the epidemiology of the 2022–2024 Mpox outbreaks, with particular attention to differences between Clade I and Clade II. In addition, a targeted search was performed on the CDC to track travel-associated cases in the United States (U.S.) in early 2025. Gray literature was evaluated based on the issuing body, publication recency, clarity of data reporting, and consistency with peer-reviewed findings. We prioritized reports from established public health authorities such as the World Health Organization (WHO), Centers for Disease Control and Prevention (CDC), and the European Centre for Disease Prevention and Control (ECDC). Sources were included only if they provided verifiable epidemiologic or surveillance data and were publicly accessible. Reports lacking methodological transparency or without clear sourcing were excluded from the final analysis. The search terms used were: Mpox (monkeypox) OR monkeypox OR Mpox AND “Clade I" OR “Clade II". The Boolean operators “AND” and “OR” were utilized to refine the results and ensure a comprehensive capture of relevant studies. Articles were eligible for inclusion if they (1) were published in English, (2) constituted primary research studies (excluding reviews) and raw epidemiological data, and (3) directly addressed the epidemiology of MPXV related to Clade I and Clade II. Articles were excluded if they (1) did not focus on epidemiological aspects of MPXV, (2) were not written in English, or (3) were review articles or other non-primary research formats. Key details, including study design, geographic scope, sample size, and specific epidemiological findings related to Clade I and Clade II, were then extracted from each selected article for analysis (Fig. A1).

## 3 Results

A total of 51 sources were included in the final analysis, comprising 14 peer-reviewed studies and 37 Gy literature documents. Peer-reviewed articles were published within the past three years and discussed epidemiological factors regarding the Mpox outbreaks. Data from each reference was extracted for consistency across epidemiological characteristics, including age, gender, geographical distribution, mode of transmission, genomic and proteomic analysis, risk factors, and case-fatality rates. Our findings are subsequently outlined and can also be visualized in Table 1, Table 2, Table 3.

**Table 1:** WHO Global Mpox Surveillance.

Report Name	Date Published	Reported Date Range	Total Number of Cases	Reported Number of New Confirmed Cases	Total Number of Deaths	Global CFR	Gender Distribution⁎	Median Age Range⁎	Mode of Transmission⁎
Situation Report #32	4/30/2024	1/1/22–3/31/24	95,226	466	185	0.2 %	M: 85,328 W: 3185	Median: 34 (IQR: 29–41)	Sexual (83.4 %)
Situation Report #33	5/31/2024	1/1/22–4/30/24	97,208	528	186	0.2 %	M: 85,759 W: 3202	Median: 34 (IQR: 29–41)	Sexual (83.6 %)
Situation Report #34	6/28/2024	1/1/22–5/31/24	97,745	646	203	0.2 %	M: 85,997 W: 3205	Median: 34 (IQR: 29–41)	Sexual (83.6 %)
Situation Report #35	8/12/2024	1/1/22–6/30/24	99,176	934	208	0.2 %	M: 87,189 W: 3221	Median: 34 (IQR: 29–41)	Sexual (83.8 %)
Situation Report #36	9/14/2024	1/1/22–7/31/24	103,048	3872	229	0.2 %	–	–	–
Situation Report #37	9/22/2024	1/1/22–8/31/24	106,310	3262	234	0.2 %	–	–	–
Situation Report #40	10/26/2024	1/1/22–9/30/24	109,699	3389	236	0.2 %	–	–	–
Situation Report #43	12/09/2024	1/1/22–10/31/24	115,101	5402	255	0.2 %	–	–	–
Situation Report #44	12/23/2024	1/1/22–12/15/24	117,663	2726	263	0.2 %	–	–	–

Table 1 Data compiled from WHO surveillance reports for MPXV Clade I and II [24,25,30,34,[44], [45], [46], [47], [48]].

⁎ Notably, the total number of cases were reported, however, individual demographic data was limited. Thus, gender distribution, median age range, and mode of transmission is only a subset of available information from the total prevalence. (CFR = Case-Fatality Rate; IQR = interquartile range; M = men; W = women).

**Table 2:** WHO African Mpox Surveillance.

Report Name	Date Published	Reported Date Range	Total Confirmed Cases	Total Suspected Cases	New Confirmed Cases	Total Confirmed Deaths	Total Suspected Deaths	Confirmed Cases in DRC	Confirmed Deaths in DRC	Confirmed Cases in Burundi	Confirmed Deaths in Burundi
Situation Report #36	9/14/2024	1/1/24–9/8/24	5759	25,093	–	32	723	–	–	–	–
Situation Report #37	9/22/2024	1/1/24–9/15/24	6201	29,342	442	32	812	–	–	–	–
Situation Report #38	9/28/2024	1/1/24–9/22/24	6603	31,427	402	32	844	5621	25	696	0
Situation Report #39	10/6/2024	1/1/24–9/29/24	6754	35,525	151	32	996	5610	25	853	0
Situation Report #40	10/13/2024	1/1/24–10/6/24	7535	36,787	781	32	998	6169	25	987	0
Situation Report #41	10/26/2024	1/1/24–10/20/24	9320	43,799	1785	34	1012	7534	25	1287	0
Situation Report #42	11/9/2024	1/1/24–11/3/24	11,148	46,794	1828	53	1081	8662	43	1726	0
Situation Report #43	12/9/2024	1/1/24–12/1/24	13,171	–	2023	57	–	9513	43	2334	1

Table 2**:** Extracted data from WHO regarding the surveillance of MPXV Clade I and II in Africa in 2024 [[30], [31], [32], [33], [34],[47], [48], [49]]. (DRC = Democratic Republic of Congo).

**Table 3:** ECDC European Mpox Surveillance.

Report Name	Date Published	Reported Date Range	Total Confirmed Cases	Total Number of Deaths	Total Confirmed Cases in Spain	Total Confirmed Cases in France	Total Confirmed Cases in Germany
*Communicable Disease Threats Report, 14–20 January 2024, Week 3*	1/19/2024	May 2022–1/12/2024	21,860	7	7752	4171	3774
*Communicable Disease Threats Report, 7–13 April 2024, Week 15*	4/12/2024	May 2022–4/8/2024	22,298	10	7960	4206	3821
*Communicable Disease Threats Report, 6–12 July 2024, Week 28*	7/12/2024	May 2022–7/6/2024	22,585	10	8084	4272	3850
*Communicable Disease Threats Report, 10–16 August 2024, Week 33*	8/16/2024	May 2022–8/15/2024	22,662	10	–	–	–

Table 3**:** Surveillance of MPXV by ECDC in Europe from 2022 to 2024 [[50], [51], [52], [53]].

### 3.1 Peer-reviewed literature

A case study from Cameroon showed that both Clade I and Clade II Mpox viruses were circulating in the country between 1970 and 2022. Among 137 suspected cases, 32 were confirmed by polymerase chain reaction (PCR) testing. Clade I cases were mainly found in the eastern regions, while Clade II was more common in the west. The median age of suspected cases was 21.5 years, and no significant difference in infection rates was found between males and females. Three deaths were reported, all linked to Clade II infections, resulting in a case fatality rate of 2.2 % [13].

More recently, in 2023, a new Clade I Mpox outbreak emerged in the eastern Democratic Republic of the Congo (DRC), with 241 suspected cases and 108 confirmed by PCR testing. The median age of confirmed cases was 22 years, with 67 % of them between the ages of 15 and 30. Over half of the infected individuals (51.9 %) were female, and 29 % identified as sex workers. None of the confirmed cases had received a smallpox vaccine, which was discontinued in the DRC in 1971 [14]. In this study, genomic analysis also revealed APOBEC3C-type mutations, particularly cytidine-to-thymidine (C → T) transitions, consistent with the action of APOBEC3 enzymes, a family of host cytidine deaminases that can introduce G-to-A hypermutations in viral genomes. These mutations, frequently identified in Clade I MPXV lineages from the DRC, represent a potential molecular mechanism driving increased human-to-human transmissibility and viral adaptation. APOBEC3 editing leaves characteristic mutational signatures that can be traced phylogenetically, allowing researchers to identify evolutionary clusters and transmission patterns. With the identification of these molecular markers, valuable insight into the rapid evolution of MPXV may help explain the emergence of novel clades with expanded transmission capabilities [12]. The implications extend beyond surveillance suggesting a possible shift in viral tropism, immune evasion potential, and diagnostic detectability, reinforcing the need for sustained genomic monitoring and real-time molecular epidemiology. Evidently, the APOBEC3C-type mutations are strongly associated with increased human-to-human transmission.

Another study found that Clade I Mpox virus contains immune evasion proteins, such as B22, F3, and Crm that may help the virus avoid detection by the body's immune system [15]. While Clade I was historically linked to animal-to-human transmission with limited spread between people, recent evidence suggests a shift toward more sustained human-to-human transmission, including through sexual contact [14,16]. Clade IIb has also been found in air samples, raising concerns about possible airborne transmission. Although Mpox mainly spreads through close physical contact, its presence in aerosols could represent an additional public health risk [16]. During the 2022 global outbreak, Clade IIb Mpox spread to over 100 countries, with most cases occurring in urban areas and among men who have sex with men (MSM) [17]. By December 14, 2023, Mpox had been reported in 116 countries, with 91,788 confirmed cases and 167 deaths worldwide [18]. Meanwhile, in the Democratic Republic of the Congo (DRC), Clade I Mpox continued to spread, with 19,919 confirmed cases and 975 deaths reported between 2023 and 2024, resulting in a case fatality rate of 4.9 % [19]. Most of these deaths (78 %) occurred in children under 15 years old, and high transmission levels were observed in the capital city, Kinshasa. In 2024, public health reports from the DRC also noted outbreaks among sex workers and MSM populations similar to the transmission patterns seen in the 2022 Clade IIb outbreak [18].

A study from several European countries found that most men who had received the smallpox vaccine during childhood still had some protection against Mpox caused by Clade II. While the level of protection varied greatly between individuals, on average, the vaccine was estimated to reduce the risk of infection by about 70 % [20].

In addition to our primary sources, recent peer-reviewed syntheses have advanced understanding of MPXV genomic evolution, offering valuable context for interpreting the current epidemiological patterns. For example, Hajjo et al. (2025) conducted a systematic review of monkeypox virus pathophysiology and genomic plasticity, emphasizing emerging mutation patterns, including APOBEC3-mediated changes that have been linked to increased human-to-human transmissibility, particularly within Clade I strains [21]. These genomic adaptations have raised concerns not only about potential immune escape but also about the reduced efficacy of historical smallpox-derived immunity in high-risk populations. Furthermore, new meta-analyses published in 2025 have correlated certain mutation profiles with differences in disease severity, reproductive number (R₀), and response to antivirals, thereby informing future vaccine and therapeutic development [22]. This evolving genomic landscape reinforces the urgency of real-time surveillance, particularly in regions like Central Africa, where Clade I lineages are rapidly diversifying in the context of limited sequencing capacity. Integrating these recent findings adds nuance to our understanding of MPXV's transmission dynamics and highlights the importance of sustained genomic monitoring as part of global public health preparedness.

### 3.2 Findings from the gray literature

#### 3.2.1 Global epidemiology and transmission trends (2022–2024)

Between January 2022 and December 2024, more than 106,000 laboratory-confirmed Mpox cases and 234 deaths were reported across 123 countries, reflecting widespread global transmission of both Clade I and Clade II Mpox virus (MPXV) strains (Table 1) [23,24]. Clade IIb remained the primary driver of the multi-country outbreak outside of Africa, particularly in the Americas and Europe, with transmission predominantly occurring among men who have sex with men (MSM) via close physical and sexual contact. Globally, most confirmed cases (96.4 %) were in men, with a median age of 34. In non-endemic countries, about 95–96 % of new infections were linked to sexual contact [25,26]. Hospitalization rates remained steady at 7–8 % across 2023 and 2024, and no significant changes in disease severity were noted [27]. During seasonal peaks, particularly around mass gatherings such as LGBTQ+ Pride events, resurgences of Clade IIb cases prompted targeted public health messaging and expanded vaccination campaigns in Europe [25]. By December 2024, the EU/EEA had reported a cumulative 23,478 confirmed Mpox cases, led by Spain, Germany, and France [26].

#### 3.2.2 Clade I transmission dynamics and pediatric impact in Central Africa

The African Region reemerged as the epicenter of global Mpox activity in 2024, primarily driven by the geographic expansion of Clade I MPXV across the Democratic Republic of the Congo (DRC), Burundi, and neighboring countries. In the DRC, both Clade Ia and Ib strains circulated, with over 21,000 suspected Mpox cases and more than 700 deaths reported by late 2024 (Table 2) [28,29]. Among these, only 37–40 % were laboratory confirmed, and positivity rates approached 55 %, underscoring significant diagnostic limitations [30]. Children aged 0–17 years represented up to 75 % of reported cases in displacement settings, such as internally displaced persons (IDP) camps in North Kivu, and carried disproportionately high mortality rates [31,32].

As mentioned previously, a novel Clade I variant with APOBEC3-type mutations was first described in South Kivu province of the DRC in mid-2023 and was associated with ongoing human-to-human transmission, coinciding with expansion to 85 % of DRC's provinces [14,25]. Children under five years accounted for 39 % of all confirmed cases as of May 2024, including 62 % of reported deaths. Children aged 1 to 4 years faced a substantially higher risk of death from Mpox compared to other age groups, with more than three times the odds of mortality. In contrast, in Burundi, although Clade Ib Mpox led to over 500 suspected and confirmed cases by August 2024 and children under five accounted for 30 % of them, no deaths were reported in this group [31]. This contrast may reflect differences in viral characteristics, healthcare access, or reporting practices across regions.

#### 3.2.3 Pediatric outcomes and risk factors

Mpox's pediatric burden, particularly in regions like the DRC has been especially severe during the 2023–2024 outbreaks. According to multiple WHO situation reports, children under five years of age accounted for nearly 39 % of confirmed Clade I cases and over 60 % of reported Mpox-related deaths in the DRC, with many of these occurring in displaced or rural populations with limited healthcare access [23,33,34]. These severe outcomes are compounded by multiple overlapping risk factors including high rates of HIV coinfection, widespread childhood malnutrition, and exposure to overcrowded, under-resourced environments, such as internally displaced persons (IDP) camps [12,14,35]. Studies have also identified diagnostic inaccessibility and exclusion from vaccination campaigns as additional barriers that place children at disproportionate risk [10,12]. While smallpox-derived MVA-BN vaccines have been used under emergency authorizations for adolescents and adults, many children under five remain unvaccinated and clinically underserved in affected regions [9,36]. These findings reinforce the urgency of pediatric-focused outbreak response and highlight the need for expanded surveillance, treatment access, and immunization strategies tailored to high-risk child populations [9,12].

#### 3.2.4 International spread and risk assessment

By late 2024, Clade I Mpox cases were confirmed outside of Africa in Germany, India, Sweden, Thailand, the United Kingdom (U.K), and the U.S. [37,38]. In the U.S., the first case of Clade I MPXV was reported in California in November 2024, followed by travel-associated cases in Georgia, New Hampshire, and New York in early 2025 [37]. Outside Africa, Clade Ib cases were also detected in Sweden and Thailand, with no secondary transmission recorded. Germany reported a small household cluster involving non-traveling contacts, including two pediatric cases [39]. Despite these introductions, the CDC assessed that the overall risk of sustained transmission in non-endemic countries remained low due to early detection and containment [37,40]. CDC modeling studies projected that Clade I Mpox outbreaks within U.S. households would likely be small, involving fewer than ten cases per cluster, and would be self-limiting in children. However, urban sexual networks posed a higher-risk scenario for transmission [41].

#### 3.2.5 Vaccination, diagnostics, and public health response

In response to Clade I expansion, the WHO granted emergency prequalification of the MVA-BN vaccine in September 2024, extending eligibility to adolescents aged 12–17 years. As of October 2024, over 265,000 MVA-BN doses had been deployed across DRC provinces [32]. The CDC and WHO also recommended two-dose JYNNEOS vaccination for individuals at increased risk, including travelers to endemic areas and those with potential sexual exposures [42,43]. In parallel, the Alinity m MPXV PCR assay was approved under WHO's emergency use listing, expanding diagnostic access in resource-limited settings. However, as of mid-2024, only 18 % of suspected Mpox cases in the DRC had been tested, suggesting that national case tallies significantly underestimate true incidence and underscoring the need for a proactive public health response [23,43].

From our gray literature search utilizing the World Health Organization (WHO), Center for Disease Control and Prevention (CDC), and European Centre for Disease Control and Prevention (ECDC) databases, our findings were extracted, and tables were created. The data published by WHO were identified within emergency situation reports that were collected from public health government agencies [24,25,30,34,[44], [45], [46], [47], [48], [49]].

Table 3 shows European Centre for Disease Control data on Mpox surveillance from 2022 to 2024. Data from this table aggregated cases from the most affected countries: Spain, Germany, and France. A low level of transmission occurred in Europe with a calculated monthly average of 116 new confirmed cases of MPXV from January 12, 2024 to August 15, 2024. Most of the confirmed cases within the table account for the 2022 outbreak. Remarkably, only an approximate 738 cases were reported over the course of 2023 [50].

From January through September 2024, Mpox transmission dynamics in the European Union and European Economic Area (EU/EEA) remained stable, with low incidence and no significant change in disease severity or transmission route. Weekly surveillance data from the ECDC consistently reported sporadic travel-associated Clade IIb cases and rare household clusters, with no indication of widespread community transmission [[50], [51], [52], [53], [54], [55], [56], [57], [58], [59]]. These trends supported repeated low-risk assessments and the continuation of targeted, rather than population-wide, interventions.

## 4 Discussion

Our analysis of MPXV epidemiology from 2022 to 2024 highlights the complex dynamics of Clade I and Clade II transmission globally, with notable geographic and demographic trends. The emergence of a new Clade I MPXV strain within the Democratic Republic of Congo (DRC) and its international spread further complicated the already concurrent Clade IIb strain, emphasizing the urgency for public health interventions [14,23,30]. This review reaffirmed trends and documented changes observed in the disease's course by examining a mixture of peer-reviewed and gray literature [17,24]. By analyzing government data and reports from public health agencies, more effective and targeted interventions may be developed in affected countries to better support disease control efforts.

This review affirmed several common demographic features among reported cases—namely, that patients tended to be male, between the ages of 20–40, and had engaged in sex work [6,23]. A large subset of cases also had a comorbid history of HIV [6,23]. In the 2022 global outbreak, Clade IIb was a rapidly infectious strain affecting urban populations and men who have sex with men (MSM), with recent evidence of airborne transmission raising additional concerns [16,23]. With MPXV reported in 116 countries and 91,788 cases by the end of 2023, the global nature of this epidemic highlighted the virus's potential for persistent international transmission [23].

While the co-circulation of Clades I and II has been ongoing in Cameroon since 1970, Clade I was historically associated with zoonotic transmission and limited human-to-human spread [13]. However, recent public health reports suggest increasing sustained sexual transmission of both clades [14,42]. Genomic and proteomic analyses indicate that Clade I MPXV transitioned from zoonotic to human-to-human transmission, with key adaptations—such as APOBEC3C-type mutations—contributing to increased infectivity [8,14,47]. Some evidence also raises concern for aerosol spread, which could broaden the range of transmission routes [17]. Continued genomic surveillance remains essential as MPXV evolves [14,24,42].

By 2024, a total of 23,313 new MPXV cases were documented, marking a 32.4 % monthly increase. Of these cases with available demographic information, most occurred in males (96.4 %), with 83.8 % linked to sex work or MSM exposure [23]. Both Clade I and II cases were frequently reported among sex workers and persons living with HIV, with Clade I posing greater concern due to its higher transmission potential [28]. Despite this, case-fatality rates remained low, suggesting lower pathogenicity in confirmed cases [24].

Clade I MPXV demonstrated rapid proliferation in Burundi and the DRC before expanding into non-endemic regions including Europe, North America, and Asia [23,29,30]. This was unprecedented for Clade I, as prior global outbreaks mainly involved Clade II [4]. In August 2024, WHO declared a Public Health Emergency of International Concern (PHEIC) due to cross-border transmission of both clades [30]. Surveillance data indicated that Africa accounted for approximately half of all new 2024 cases, with the DRC and Burundi comprising nearly 90 % of the continent's total (Table 2). The DRC alone accounted for ∼80 %, reinforcing its status as an endemic hotspot. Biweekly reports were used to calculate a 14.4 % average increase in confirmed cases, with inadequate supplies for laboratory testing, or stockouts. Fig. A2 visualizes the cumulative confirmed cases within the African region during late 2024 [[30], [31], [32], [33], [34],[47], [48], [49]]. With testing rates at just 18 % of suspected Mpox cases in the DRC, true incidence is likely significantly underestimated [23]. Investment in point-of-care diagnostics, laboratory infrastructure, and healthcare worker training is essential to improve surveillance and response capacity. The 3I model, previously applied in high-risk healthcare settings, offers a useful template for responding to Clade I resurgence in endemic regions. Marty et al. (2024) emphasize the need to update frontline guidance as transmission patterns shift and viral evolution accelerates [12].

In the DRC in 2024, children under 5 made up 39 % of confirmed Mpox cases and 62 % of deaths. In camps for displaced people, up to 75 % of cases were in children. Young children aged 1–4 were over three times more likely to die from the disease than others, showing a critical need for better testing, vaccines, and care for children [31,32]. These findings highlight the importance of addressing health inequities in global public health planning.

Additionally, the integration of recent comprehensive reviews substantially enriches the scope of this review by broadening the discussion beyond surveillance data to encompass critical dimensions of vaccine equity, diagnostic infrastructure, and regulatory preparedness. Yadav et al. offer detailed insights into clinical management, diagnostic advancements, and therapeutic approaches, while Tiwari et al. examine the policy frameworks, governance challenges, and global coordination mechanisms that shaped the Mpox response across different health systems [9,10]. Together, these sources provide essential context for interpreting our findings, particularly in relation to persistent gaps in outbreak preparedness and response in low-resource settings. Their inclusion allows for a more nuanced understanding of how scientific progress, policy action, and operational limitations converge to influence disease control efforts on a global scale.

In 2022, Europe especially Spain, France, and Germany were stricken by Mpox. However, cases dropped in 2023 and 2024, with only 738 cases reported in 2023 and 116 new cases between January and August 2024 [26]. This decline is likely due to early public health actions and targeted education efforts [38]. A European study found that childhood smallpox vaccines offered about 70 % protection against Clade II Mpox, though the level of protection varied [20]. None of the people infected in 2023–2024 had received this vaccine, which has led public health agencies to stress the need for expanded vaccination programs for high-risk groups [20].

Furthermore, in 2024, EU/EEA transmission remained stable and sporadic, largely tied to international travel or localized clusters without broader community spread [26,27,39]. These trends reflect the success of targeted interventions and underscore the need for sustained public health vigilance even in regions with low case counts.

Discrepancies in transmission appear to correlate with disparities in public health resources. Countries in Africa reported widespread stockouts and greater rural burden compared to better-resourced urban regions [23]. International spread occurred via air travel to non-endemic countries such as Sweden, India, Germany, the U.K., and the United States (U.S.). As seen in Fig. A3, all cases were effectively contained through contact tracing and isolation protocols, which were promptly enacted by public health officials [28,56,59]. Key preventative measures, including PCR testing, contact tracing, quarantine, and promotion of herd immunity can overall help reduce transmission among vulnerable populations [18]. To address the broader biomedical and ecological context of Mpox, we expand our framing to consider how viral evolution, host vulnerability, and health system capacity interact to shape outbreak patterns. The emergence of APOBEC3-driven mutations, for example, may enhance transmissibility and challenge existing vaccine protection. In Central Africa, the disproportionate burden among children reflects intersecting factors, such as immunologic immaturity, malnutrition, displacement, and limited diagnostic access. These findings underscore the need for a One Health approach that integrates zoonotic spillover risks, environmental change, and structural health disparities. By situating surveillance and equity gaps within this interconnected framework, we aim to strengthen the translational relevance of epidemiological data for global health preparedness [19].

#### 4.1.1 Pediatric vulnerability and public health preparedness

The 2024 resurgence of Clade I Mpox in Central Africa has revealed an urgent and disproportionate impact on pediatric populations, particularly children under five years of age. In the DRC, this group accounted for nearly 40 % of confirmed cases and over 60 % of reported deaths, with many affected individuals residing in internally displaced persons (IDP) camps where malnutrition, HIV prevalence, and delayed access to care further elevate risk [60]. Despite this, pediatric-specific surveillance and clinical protocols remain fragmented. Child-appropriate diagnostics are limited, and emergency vaccine rollouts have not adequately covered this vulnerable group. To mitigate preventable mortality and ensure equitable outbreak response, national and global health systems must integrate Mpox planning into routine childhood immunization, develop protocols for pediatric triage and case management, and expand access to antiviral treatments such as tecovirimat and vaccinia immune globulin in suspension or age-adjusted formulations [61]. These actions are especially critical in conflict-affected and resource-limited regions, where children face multiple, compounding health threats (Table 4).

**Table 4:** Recommended Action Areas for Pediatric Mpox Preparedness and Response.

Domain	Recommendation
Diagnostics	Deploy pediatric-compatible PCR kits to subnational labs; establish testing in IDP clinics.
Treatment	Stock pediatric formulations of tecovirimat (e.g., suspension) and vaccinia immune globulin (VIG).
Vaccination	Pre-authorize emergency MVA-BN use in children <5 years in outbreak zones; train staff on delivery.
Surveillance	Implement syndromic surveillance in schools, IDP camps, and pediatric inpatient units.
Integration	Add Mpox to EPI (Expanded Programme on Immunization) planning for routine and outbreak response.
Health Systems	Establish mobile care teams for outbreak zones with high child density and weak infrastructure.

Footnotes: IDP: Internally Displaced Persons, MVA-BN: Modified Vaccinia Ankara–Bavarian Nordic.

## 5 Future Directions

To effectively address the evolving challenges of MPXV transmission, the most urgent priority is the expansion of diagnostic testing capacity in resource-limited settings such as the Democratic Republic of the Congo (DRC), where current confirmation rates remain below 20 %. Improved diagnostics are foundational for surveillance, case management, and outbreak containment. Once diagnostic gaps are addressed, enhanced genomic surveillance should follow to detect emerging mutations, such as APOBEC3-type changes that may increase human-to-human transmissibility. Simultaneously, pediatric-focused vaccination strategies must be implemented, given the disproportionately high burden and mortality among children under five, especially in displacement and conflict-affected regions. Tailored public health communication is also needed to reduce stigma and improve outreach among high-risk groups, including MSM and sex workers. Finally, strengthened coordination between global health organizations and national public health authorities is essential to standardize outbreak reporting, streamline emergency responses, and ensure equitable access to vaccines and therapeutics across both endemic and non-endemic regions.

## 6 Strengths and limitations

This review offers a comprehensive synthesis of recent peer-reviewed literature and gray sources on the evolving epidemiology of Mpox from 2022 to 2024, providing timely insights into clade-specific transmission patterns, geographic spread, and public health responses. A key strength lies in the integration of official surveillance data and situational reports from major global health organizations, which helped capture real-time developments often missed in academic literature. The inclusion of data from both endemic and non-endemic regions further enhances the global relevance of findings. However, limitations include variability in data quality across sources, especially within gray literature, where differences in reporting standards, methodological transparency, and peer review may introduce inconsistencies. Additionally, the rapidly changing nature of Mpox outbreaks may limit the generalizability of some findings over time. Despite these constraints, the review highlights critical trends and gaps that can inform future research, surveillance, and outbreak preparedness.

## 7 Conclusion

This comprehensive review of MPXV outbreaks from 2022 to 2024 highlights critical shifts in the global epidemiology of both Clade I and Clade II lineages. Clade IIb was responsible for an unprecedented international outbreak beginning in 2022, with more than 91,000 confirmed cases reported across 116 countries by the end of 2023. Transmission occurred predominantly through close physical and sexual contact, particularly among men who have sex with men, with a significant proportion of cases occurring in individuals co-infected with HIV. Despite its wide geographic spread, the Clade IIb outbreak was associated with relatively mild clinical outcomes and a low case-fatality rate.

In contrast, Clade I MPXV demonstrated a resurgence centered in Central Africa, especially in the Democratic Republic of the Congo and neighboring regions. With disproportionately high mortality in unvaccinated children under 15 years, this variant spread rapidly within endemic zones and led to isolated introductions in non-endemic countries. These events culminated in the declaration of a Public Health Emergency of International Concern in 2024 by the World Health Organization.

Key findings from this review emphasize the urgent need for expanded genomic surveillance, improved diagnostic access, and equitable vaccine distribution—especially in low-resource settings. Persistent disparities in public health infrastructure, testing capacity, and vaccine coverage have contributed to regional differences in disease burden and transmission dynamics. As MPXV continues to evolve and cross borders, sustained global coordination and data standardization will be essential to mitigating future outbreaks.

The following are the supplementary data related to this article.

Supplementary Fig. S1 Fig. A1. Flow diagram summarizing the literature search strategy and selection process for Mpox (MPXV) epidemiology studies from 2022 to 2024, with a focus on Clade I and Clade II distinctions. Supplementary Fig. S1

Supplementary Fig. S2 Fig. A2. Cumulative Mpox cases in Africa from September to December 2024, highlighting a consistent rise, with notable increases in later months [[30], [31], [32], [33], [34],[47], [48], [49]]. Supplementary Fig. S2

Supplementary Fig. S3 Fig. A3. Geographic distribution of confirmed Clade I Mpox cases in 2024, highlighting endemic regions, local transmission, and travel-related importation. The map illustrates transmission pathways and the spread of cases across multiple continents based on WHO surveillance data [28,56,59]. Supplementary Fig. S3

## CRediT authorship contribution statement

**Baylor Akhavan:** Visualization, Validation, Supervision, Project administration, Methodology, Investigation, Formal analysis, Data curation, Conceptualization, Writing – review & editing, Writing – original draft. **Abu-Bakr Ahmed:** Visualization, Project administration, Methodology, Investigation, Conceptualization, Writing – review & editing. **Wilson To:** Validation, Resources, Project administration, Formal analysis, Writing – review & editing, Writing – original draft. **Samir Alkhouri:** Visualization, Investigation, Writing – review & editing. **Kavita Batra:** Supervision, Methodology, Writing – review & editing, Writing – original draft.

## Ethics approval and consent to participate

Not applicable. This review article did not involve any studies with human participants or animals performed by any of the authors.

## Funding

No external funding was used to support this research.

## Declaration of competing interest

The authors declare no conflict of interest.

## Data Availability

Data will be made available on request.
