# Supplementary material for: From forest to frontline: A comprehensive review of Mpox's global leap and viral evolution (2022–2024)
Source: One Health. 2025 Oct 3;21:101232. doi: 10.1016/j.onehlt.2025.101232 (PMC12538092; doi:10.1016/j.onehlt.2025.101232)
Supplement: Supplementary Fig. S1 — Fig. A1. Flow diagram summarizing the literature search strategy and selection process for Mpox (MPXV) epidemiology studies from 2022 to 2024, with a focus on Clade I and Clade II distinctions. [file mmc1.pdf]

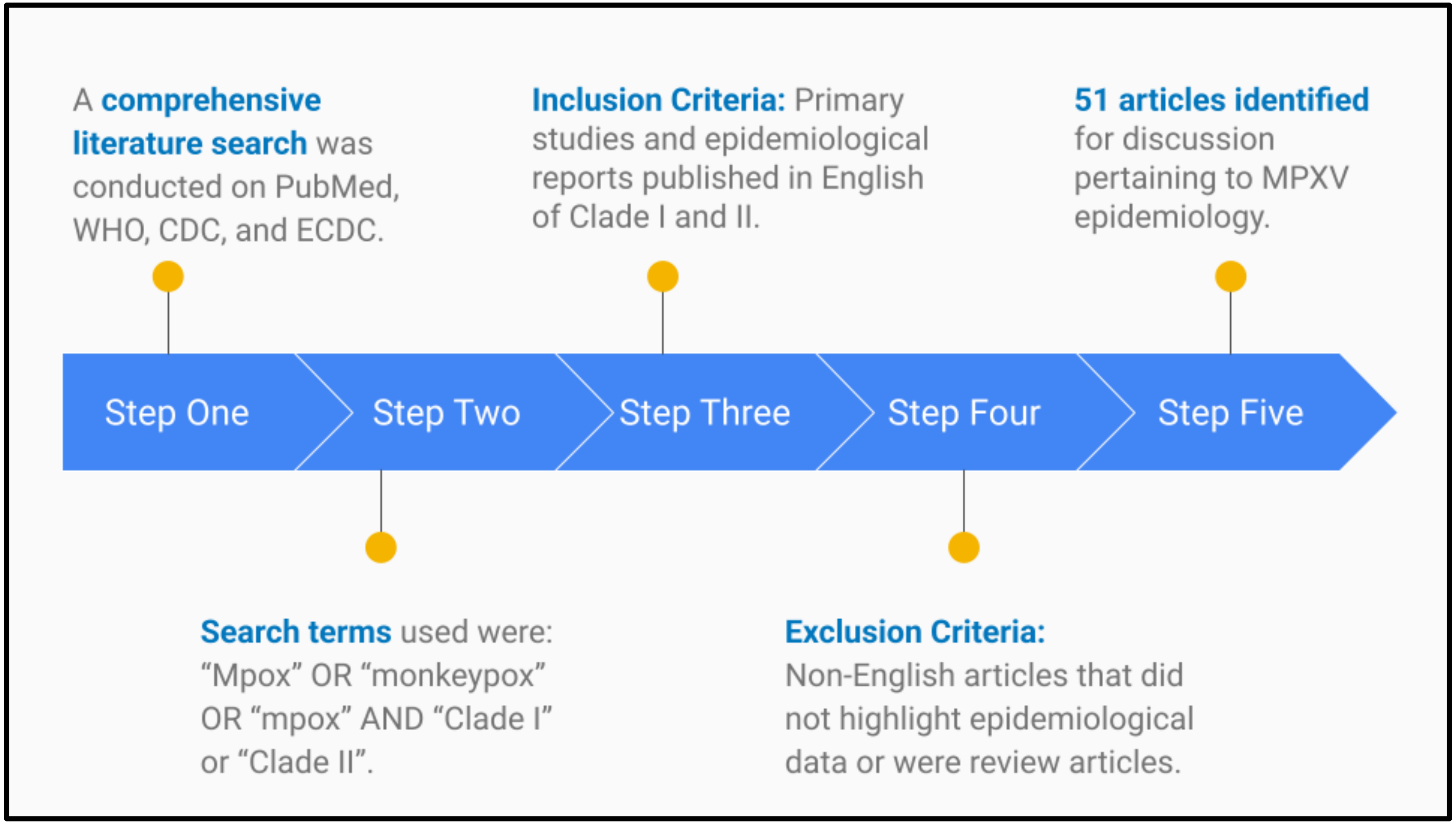
A **comprehensive literature search** was conducted on PubMed, WHO, CDC, and ECDC.
Inclusion Criteria: Primary studies and epidemiological reports published in English of Clade I and II.
51 articles identified for discussion pertaining to MPXV epidemiology.
Step One
Step Two
Step Three
Step Four
Step Five
Search terms used were: "Mpox" OR "monkeypox" OR "mpox" AND "Clade I" or "Clade II".
Exclusion Criteria: Non-English articles that did not highlight epidemiological data or were review articles.
