# Supplementary figures and images for: From forest to frontline: A comprehensive review of Mpox's global leap and viral evolution (2022–2024)
Source: One Health. 2025 Oct 3;21:101232. doi: 10.1016/j.onehlt.2025.101232 (PMC12538092; doi:10.1016/j.onehlt.2025.101232)

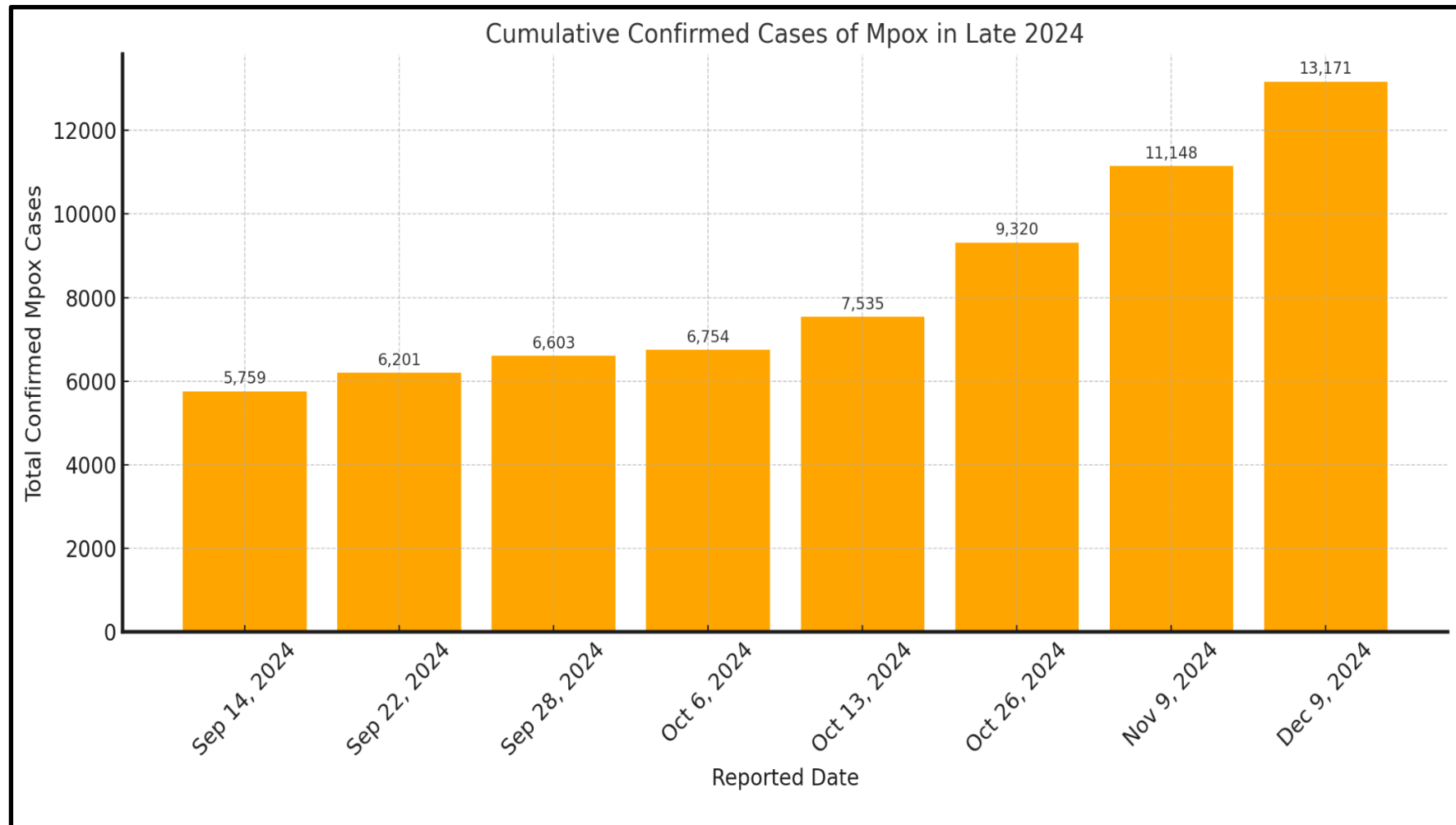

Supplement: Supplementary Fig. S2 — Fig. A2. Cumulative Mpox cases in Africa from September to December 2024, highlighting a consistent rise, with notable increases in later months [[30], [31], [32], [33], [34],[47], [48], [49]]. [file mmc2.pdf]

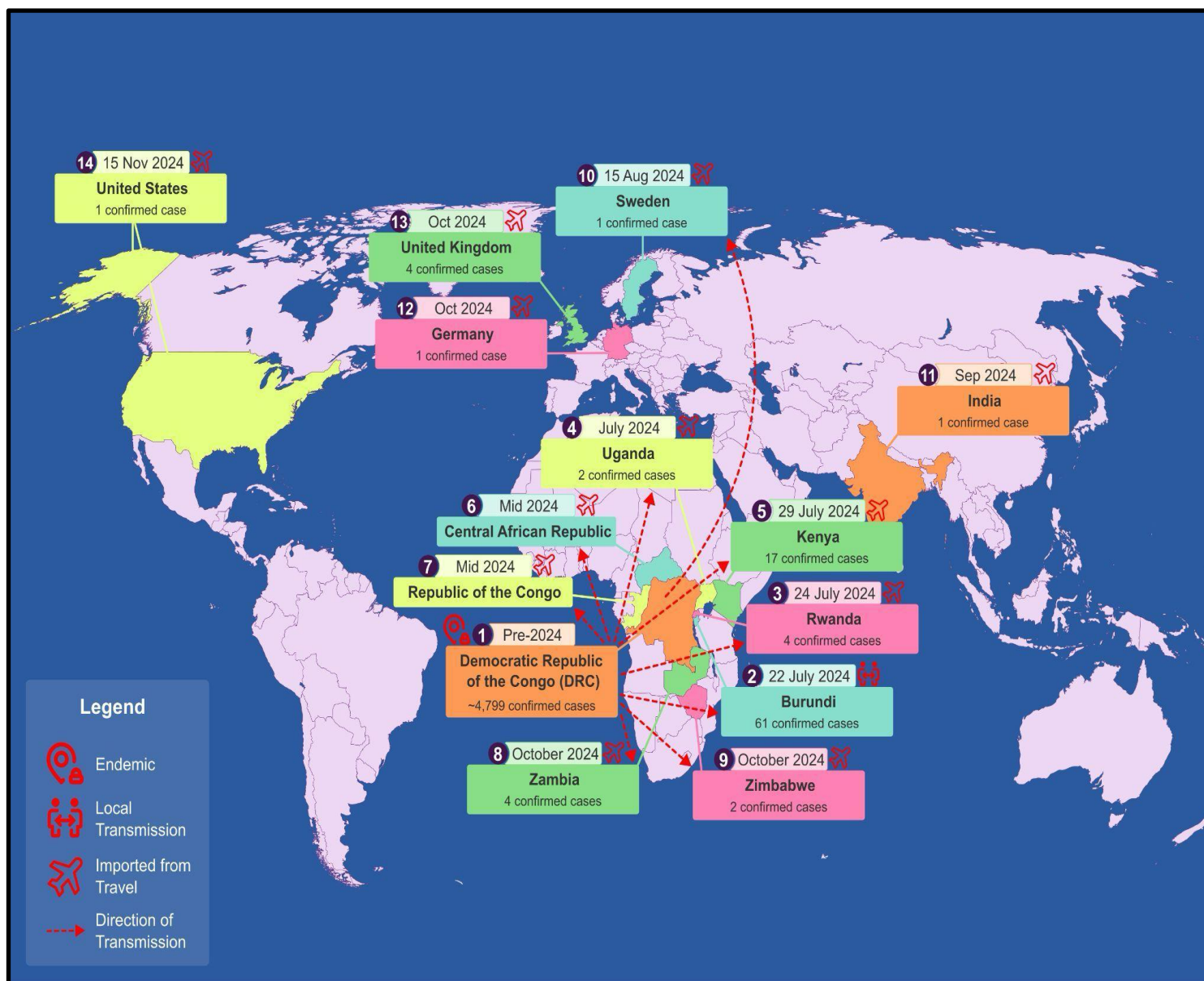

Supplement: Supplementary Fig. S3 — Fig. A3. Geographic distribution of confirmed Clade I Mpox cases in 2024, highlighting endemic regions, local transmission, and travel-related importation. The map illustrates transmission pathways and the spread of cases across multiple continents based on WHO surveillance data [28,56,59]. [file mmc3.pdf]
